# A hidden cause of oxalate nephropathy: a case report

**DOI:** 10.1186/s13256-021-02732-6

**Published:** 2021-03-08

**Authors:** Tala Mahmoud, Elias C. Ghandour, Bernard G. Jaar

**Affiliations:** 1 Faculty of Medicine, University of Balamand, El-Koura, Lebanon; 2 Department of Medicine, Division of Nephrology, Johns Hopkins School of Medicine, Baltimore, MD USA; 3 Nephrology Center of Maryland, Baltimore, MD USA; 4 Welch Center for Prevention, Epidemiology and Clinical Research, Johns Hopkins University, Baltimore, MD USA; 5 Department of Epidemiology, Johns Hopkins Bloomberg School of Public Health, Baltimore, MD USA

**Keywords:** Oxalate, Oxalosis, Hyperoxaluria, Chronic kidney disease, End-stage kidney disease, Acute kidney injury, Nephrolithiasis, Diet, Vegetables

## Abstract

**Background:**

Oxalate nephropathy is a rare disorder that can result in acute kidney injury (AKI) and progresses to end-stage kidney disease (ESKD). The causes can be either primary or secondary. Primary hyperoxaluria includes a group of hereditary disorders with enzymatic defects in the glyoxylate pathway, resulting in decreased oxalate metabolism. Secondary hyperoxaluria, often overlooked can result from increased intestinal absorption, nutritional deficiencies, decreased fluid intake, impaired excretion, and increased dietary consumption of oxalate.

**Case presentation:**

We present a Caucasian case of acute oxalate induced nephropathy associated with consumption of large quantities of green vegetables in a patient with chronic kidney disease (CKD). Imaging study showed no evidence of kidney stone, but a kidney biopsy revealed acute tubular injury, tubular atrophy, interstitial fibrosis, and dense tubular deposition of calcium oxalate crystals. Upon further questioning the patient, we learned that in the months prior to presentation, he had very significantly increased his consumption of green vegetables. Because of no clinical improvement, the patient was initiated and maintained on hemodialysis.

**Conclusion:**

This report illustrates a case of acute oxalate nephropathy in the setting of very high dietary consumption of oxalate-rich foods in a patient with advanced CKD. Special attention should be given to the secondary causes of hyperoxaluria in patients with predisposing conditions such as CKD.

## Background

Oxalate is a simple dicarboxylic acid, often found in a variety of plants and leafy vegetables [1]. It is produced exogenously from consumption of oxalate-rich foods or endogenously from the breakdown of ascorbic acid and amino acids [2]. Major dietary sources of oxalate include leafy green vegetables (e.g., spinach), chocolate, rhubarb, beets, chard, tea, nuts and wheat bran [3]. The absorption of oxalate occurs in the stomach, small intestine and large intestine; mediated by both paracellular and transcellular reuptake [4]. Excretion of absorbed and endogenously produced oxalate occurs in the urine in an unchanged manner due to insignificant metabolism in the human body [1]. Several factors may affect the absorption and excretion of oxalate, contributing to the development of hyperoxaluria. The presence of excess oxalate in the urine may lead to the development of nephrocalcinosis, nephrolithiasis or oxalate nephropathy [5]. Here, we report a case of biopsy-proven acute oxalate nephropathy.

## Case presentation

A 78-year-old Caucasian man with underlying Alzheimer’s disease presented to the emergency department with a serum creatinine level of 6.52 mg/dL from a baseline value of 1.9 mg/dL 3 months prior to presentation. Patient had a known history of benign prostatic hypertrophy with elevated post-void residual volume, thus in the emergency department a Foley catheter was placed to rule out obstructive uropathy. He also had previously experienced left hydronephrosis due to nephrolithiasis which were secondary to calcium oxalate, and he underwent lithotripsy at that time. A bedside kidney ultrasound performed in the emergency department showed no stone or hydronephrosis.

According to his wife, the patient had poor appetite but was reportedly consuming adequate amounts of liquids. The patient’s blood pressure was also monitored at home and there were no reports of hypotension. The patient has not had episodes of nausea, vomiting or diarrhea. There had been no consumption of nonsteroidal anti-inflammatory drugs, and no new medications were recently added. He had no malabsorptive symptoms and no gastro-intestinal surgeries. He had no family history of kidney disease or kidney stones.

Physical examination was unremarkable. Laboratory data were obtained and on presentation, the patient’s serum creatinine concentration was 6.52 mg/dL, corresponding to an estimated glomerular filtration rate (GFR) of 7 mL/min/1.73 m^2^ from a baseline of 34 mL/min/1.73 m^2^ (Table 1). Urinalysis showed hazy yellow urine with occasional bacteria and moderately increased proteinuria (100 mg/dL). The patient was admitted to the hospital for further evaluation and management of his AKI on CKD. A serological work-up was performed and results were unrevealing. Further, urine and serum electrophoresis showed no monoclonal gammopathy. In addition, and to avoid further calcium load, calcium acetate was discontinued, and the patient was started on IV fluid hydration and oral Sevelamer carbonate 800 mg taken with each meal for his hyperphosphatemia.

**Table 1.** Basic metabolic panel and complete blood count on day of admission

	Result	Units	
Sodium	129	mEq/L	Low
Potassium	4.4	mEq/L	
Chloride	97	mEq/L	
Bicarbonate	21	mEq/L	
Blood urea nitrogen	55	mg/dL	
Creatinine	6.52	mg/dL	High
Estimated GFR	7	mL/min/1.73 m^2^	Low
Glucose, random	144	mg/dL	High
Calcium	8.2	mg/dL	Low
Phosphorus	5.5	mg/dL	High
Albumin	3.6	g/dL	
Creatine kinase	144	units/L	
Hemoglobin	10.3	g/dL	Low
Hematocrit	30.1	%	
Leukocytes	6.5	10^9^/L	
Platelet count	161	10^9^/L	

**Fig. 1a:**
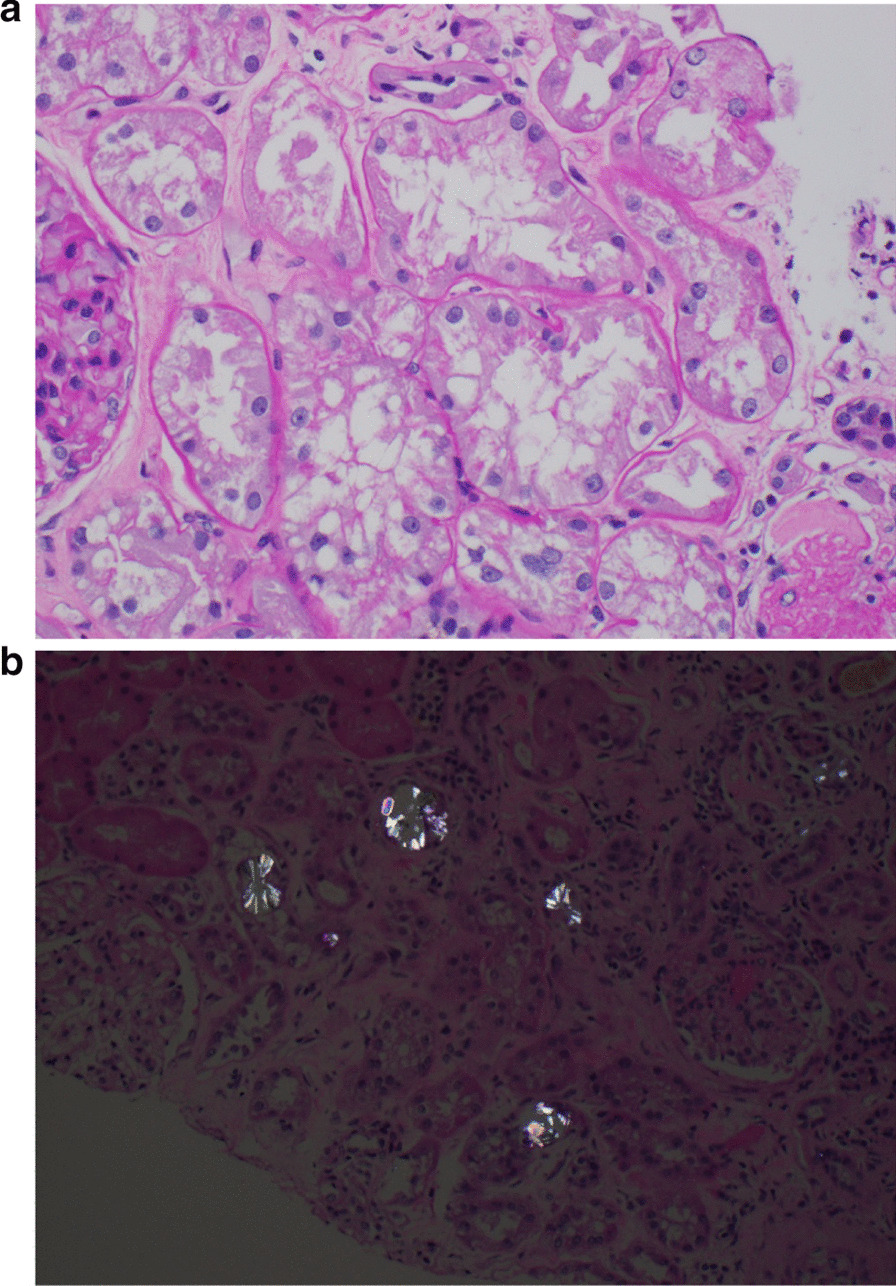
Periodic Acid-Schiff (PAS) stain magnification 400×—acute tubular injury. **b** Hematoxylin eosin stain magnification 200×—Oxalate crystals under polarized light

To better understand the cause of the patient’s AKI, a left sided kidney biopsy was performed. Light microscopy showed numerous colorless, bifringent intratubular crystals, consistent with calcium oxalate associated with focal edema and tubular injury and mixed interstitial inflammation. Tubular atrophy and interstitial fibrosis involved 50% of the cortex and 8 of 26 glomeruli were obsolescent with focal periglomerular fibrosis on light microscopy (Fig. 1a, b). Immunofluorescence revealed non-specific staining of tubular and glomerular membranes for albumin, immunoglobulin G, kappa and lambda light chains. Electron microscopy showed variable and irregular thickening of the glomerular basement membranes, consistent with ischemic change. Loss of brush border was also noted. A diagnosis of acute tubular injury with extensive calcium oxalate crystal deposition was made.

Following the kidney biopsy findings, we further inquired about the patient’s diet, and found that several weeks prior to his illness, the patient started consuming a larger amount of leafy green vegetables than usual. The patient had significantly increased his consumption of leafy green vegetables and was not aware of the excess level of oxalate present in his diet. This might explain a much higher source of dietary oxalate compared to his regular prior diet and could be the cause of his oxalate nephropathy given the absence of other explanations for his worsening kidney function. The patient was advised to drink plenty of fluids and avoid any nephrotoxic agents (such as IV contrast dye, aminoglycosides, and nonsteroidal anti-inflammatory drugs). He was also maintained on IV hydration. However, despite medical management, the patient’s kidney function continued to deteriorate. Therefore, the decision was made to start the patient on hemodialysis. Table 2 describes the renal function tests of the patient. The initiation of hemodialysis on day 5 after admission resulted in an improvement of the renal function tests. Hemodialysis was then held for a period to check for any evidence of renal function recovery. However, the patient’s kidney function continued to worsen and thus the decision was made to maintain the patient on hemodialysis.

**Table 2:** Trend in renal function panel

Time from admission	Day 4	Day 5 (First hemodialysis session)	Day 6	Day 14 (Second hemodialysis session)	Day 18
Blood urea nitrogen (mg/dL)	86	92	59	90	70
Estimated GFR (mL/min/1.73 m^2^)	5	5	8	5	6
Creatinine (mg/dL)	8.67	8.88	6.28	8.98	8.10

*GFR* Glomerular filtration rate

## Discussion

Our patient was found to have extensive calcium oxalate crystals deposition in addition to acute tubular injury. He required initiation of intermittent hemodialysis as he showed no signs of kidney function recovery. The causes of hyperoxaluria can be primary or secondary. Primary hyperoxalurias are a group of rare autosomal recessive (AR) disorders of glyoxylate metabolism, resulting in overproduction of oxalate. Primary Hyperoxaluria type 1, the most severe form, is caused by a deficiency in alanine-glyoxylate aminotransferase (*AGT*)*,* a liver-specific peroxisomal enzyme responsible for the transamination of glyoxylate to glycine. Primary Hyperoxaluria type 2, a less severe form, is caused by a deficiency in glyoxylate reductase-hydroxypyruvate reductase (*GRHPR*), a primarily intrahepatic enzyme responsible for reduction of glyoxylate to glycolate and hydroxypyruvate to d-glycerate. Primary Hyperoxaluria type 3, the least severe form, is caused by a deficiency in 4-hydroxy-2-oxo-glutarate aldolase (*HOGA*), a liver specific mitochondrial enzyme responsible for metabolism of hydroxyproline [6, 7]. Primary hyperoxaluria can occur at almost any age, ranging from birth until the sixth decade of life [6]. However, the median age of onset is 5.5 years [8]. The clinical manifestations range from nephrocalcinosis during infancy to occasional stone formation during adulthood and diagnosis is established by genetic testing [6].

Supportive management includes increasing fluid intake, decreasing oxalate in the diet, urine alkalization, and taking calcium binders that reduce precipitation of calcium oxalate stones [7, 9, 10]. Pyridoxine was found to be beneficial in patients with Primary Hyperoxaluria type 1, through reducing excretion of urinary oxalate [11]. Definite curative treatment in Primary Hyperoxaluria type 1 patients is combined liver/kidney transplantation; however, the role of liver transplantation in patients with Primary Hyperoxaluria type 2 is still unclear. Pre-emptive combined liver and kidney transplantation should be considered in Primary Hyperoxaluria patients with GFR less than 30 mL/min per 1.73 m^2^ [12].

In contrast, secondary hyperoxaluria may occur due to increased absorption of oxalate, nutritional deficiencies, decreased oxalate excretion, or increased dietary oxalate consumption.

The first mechanism leading to secondary hyperoxaluria is enteric hyperoxaluria. Dietary calcium normally binds to oxalate in the intestines. In the setting of fat malabsorption, calcium is sequestered by the luminal fat, resulting in increased free oxalate in the intestines and thus increased reabsorption [13]. This can arise in patients with a history of bariatric surgery, pancreatic insufficiency, crohn’s disease, celiac sprue, cystic fibrosis, orlistat and octreotide use, and *Clostridioides difficile* colitis [14–21]. The second mechanism leading to secondary hyperoxaluria is nutritional deficiencies. Thiamine and pyridoxine are essential cofactors in the glyoxylate pathway, and a deficiency results in increased oxalate formation [22]. The third mechanism occurs in the setting of significantly decreased fluid intake. Lower fluid intake leads to lower urine output, hence promoting the formation of oxalate stones through increased concentration of urine oxalate. The fourth mechanism is decreased oxalate excretion. In chronic kidney disease, lower GFR is associated with elevated plasma oxalate levels [23]. The degree of kidney function impairment is often correlated with the serum oxalate concentration [24]. This is because oxalate is removed from the body almost entirely by glomerular filtration and by secretion at the level of the proximal tubule [25–30]. When plasma oxalate level exceeds its plasma supersaturation level of 30 μmol/L, this results in its deposition in several extra-renal tissues, including: retina, myocardium, blood vessel walls, bone, skin and central nervous system [5]. Finally, increased dietary oxalate may lead to secondary hyperoxaluria, particularly in patients with underlying CKD. Oxalate is present in a wide variety of leafy green vegetables and fruits. Table 3 includes a list of common foods high in oxalate. In addition, vitamin C and ethylene glycol are important sources of oxalate precursors. Recent studies suggest that about 50% of urinary oxalate is derived from the diet [31, 32]. Dietary oxalate is absorbed in the intestines by SLC26 family of transporters, via both paracellular and transcellular pathways [33]. The intestinal absorption of oxalate is affected by the bioavailability of the ingested oxalate [34].

**Table 3:** Oxalate content (mg/100 g) in some common foods [3]

Food	mg/100 g
Tea	1150
Spinach	970
Rhubarb (raw)	805
Cocoa	700
Beet leaves	610
Rhubarb (stewed)	460
Red beetroot	275
Parsley	170
Coffee	100
Potato	80
Cabbage	60
Blackcurrant	50
Tomato	20
Apple	15
Lettuce	12

Management of secondary hyperoxaluria involves: (1) maintaining a low-oxalate and high-calcium diet, which helps prevent hyperoxaluria, (2) increasing fluid intake (>3 L/1.73 m^2^), (3) supplementation with calcium or other oxalate binders and bile acid sequestrants, which decreases permeability of oxalate in the intestines, (4) consumption of probiotics that contain oxalate-degrading bacteria, however recent studies have not proven its definite efficacy [12, 35].

The disease spectrum of hyperoxaluria ranges from nephrocalcinosis, nephrolithiasis to oxalate nephropathy. Nephrocalcinosis and nephrolithiasis may remain asymptomatic, with slow CKD progression, often discovered incidentally on radiographic imaging. Acute oxalate nephropathy is characterized by AKI and often progresses to ESKD. In such event, renal replacement therapy may be required and if concern for primary hyperoxaluria is present, combined liver and kidney transplantations may need to be considered [5, 36, 37].

## Conclusion

In summary, our patient with underlying CKD (stage G3B) developed acute oxalate nephropathy leading to ESKD from secondary oxalosis due to excessive consumption of oxalate-rich foods and likely decreased fluid intake. Patients with predisposing conditions such as CKD have a higher risk of developing oxalate-induced nephropathy. In addition, our case illustrates the importance of a kidney biopsy in determining the etiology of AKI when the etiology remains elusive. Therefore, in addition to the typical work-up, special attention should be warranted to the dietary habits of patients at high risk, such as those with underlying CKD.

## Data Availability

Not applicable.

## Abbreviations

CKD: Chronic kidney disease
ESKD: End-stage kidney disease
AKI: Acute kidney injury
GFR: Glomerular filtration rate
AGT: Alanine-glyoxylate aminotransferase
GRHPR: Glyoxylate reductase-hydroxypyruvate reductase
HOGA: 4-Hydroxy-2-oxo-glutarate aldolase
